# The association of serum uric acid levels in psoriasis patients: A systematic review and network meta-analysis

**DOI:** 10.1097/MD.0000000000017643

**Published:** 2019-11-01

**Authors:** Yuan Yuan, Ming Liu, WenHong Liu, Hua Du

**Affiliations:** a Gansu University of Traditional Chinese Medicine; b The 940th Hospital of Joint Logistics Support Force of Chinese People, Lanzhou; c Wuwei Hospital of Traditional Chinese Medicine, Wuwei, China.

**Keywords:** hyperuricemia, network meta-analysis, psoriasis, serum uric acid, systematic review

## Abstract

**Background:**

Current research has proved that psoriasis is associated with serum uric acid (SUAC) levels. Our purpose is to clarify SUAC levels and the incidence of hyperuricemia in psoriasis patients, and to compare SUCA levels in different groups’ psoriasis patients.

**Methods:**

We plan to search 7 electronic bibliographic databases (PubMed, Embase, Cochrane, and 4 Chinese databases) from inception to August 2019. Literatures selection and data collection will be performed independently by 2 authors. The Newcastle–Ottawa scale will be used to assess the methodologic quality and bias of included studies. Firstly, standard pairwise meta-analysis will be used to examine the considered data synthesis. Secondly, if the identified studies appear sufficiently similar within and across the different comparisons between different groups of psoriasis patients, we will estimate SUAC levels using network meta-analysis in different age and ethnicity psoriasis patients. Mean difference, risk ratio, and 95% confidence intervals will be used to assess the SUAC levels and the incidence of hyperuricemia in psoriasis patients. The software of Stata and WinBUGS will be used to calculations.

**Results:**

The results will be published in a peer-reviewed journal.

**Conclusion:**

Our study will compare SUCA levels in different groups’ psoriasis patients through network meta-analysis, and we believe our job is very meaningful.

**Ethics and dissemination:**

Our study is a secondary study of the existing literature. So, ethical and dissemination approval is not required.

## 1 Introduction

Psoriasis is a genetically related immune-mediated disease manifesting in the skin or joints or both.^[1]^ In addition, psoriasis causes is often thought to be related to climate, sun exposure, and ethnicity. Clinically, the most common symptoms are pain, itch, and bleeding.^[2]^ According to different clinical features, psoriasis is divided into 5 types: plaque psoriasis, guttate (droplet) or eruptive psoriasis, inverse psoriasis, pustular psoriasis, and erythrodermic psoriasis. According to the survey, the prevalence of psoriasis is also high. In North America and Europe, psoriasis prevalence is about 2%.^[3]^ The prevalence increases roughly linearly throughout the life cycle, from 0.12% at 1 year of age to 1.2% at 18 years of age.^[1,4]^ Like other skin disorder, psoriasis also imposes both physical and psychologic burdens. Several comorbid diseases (such as hypertension, diabetes, cardiovascular diseases, etc) often increase patients’ burden. As psoriasis imposes a huge burden on patients, families, and society, it has attracted the attention of the World Health Organization.^[5]^

Uric acid is the final product of purine metabolism. Hyperuricemia was defined as serum uric acid (SUAC) above 7.7 mg/dL in men and above 6.6 mg/dL in women.^[6]^ Current study showed that hyperuricemia is not only directly related to gout, but also to cardiovascular disease (especially sudden cardiac death), hypertension, diabetes, metabolic diseases, etc.^[7–9]^ However, the relationship between psoriasis and SUAC was once controversial.^[10–12]^ In 2016, a systematic review showed that psoriasis and hyperuricemia were related (either ethnicity or region dependent).^[13]^ However, this review including original research is not enough, and new research results have been published in recent years.^[14–16]^

In view of that, our research has 3 purposes: SUAC levels in patients with psoriasis compared with others; the risk of hyperuricemia in patients with psoriasis in all study; SUAC levels in patients with psoriasis in different populations (age, ethnicity, etc) through direct and indirect comparison.

## 2 Methods

### 2.1 Study registration

The content of this protocol follows the Preferred Reporting Items for Systematic Review and Meta-analysis Protocols (PRISMA-P) recommendations.^[17]^ The review is registered in International Prospective Register of Systematic Reviews (PROSPERO),^[18]^ with the registration number CRD42019140342. If protocol amendments occur, the dates, changes, and rationales will be tracked in PROSPERO.

### 2.2 Eligibility criteria

Studies will be included in our review if they meet all of the criteria mentioned in the following sections.

#### 2.2.1 Types of participants and exposure

Patient with psoriasis will be included. There are no limitations on the participant's age, gender, or nationality. In control group, participants are able-bodied person or others that with the disease that clearly does not increase SUCA levels or cause hyperuricemia.

#### 2.2.2 Types of study

Human-only observational studies that include descriptive studies and analytical studies, such as cohort studies, case–control studies, cross-sectional studies, etc, all will be included. Case reports, case series, review articles, letters, and commentaries will be excluded.

#### 2.2.3 Types of outcome

The primary outcomes will include the difference in mean SUAC levels between patients with psoriasis and others. Second outcomes will include the correlation of psoriasis with the risk of hyperuricemia for every study.

### 2.3 Search strategy

We will search the following 7 electronic bibliographic databases from inception to August 2019: PubMed, Embase, the Cochrane Central Register of Controlled Trials (CENTRAL), the China National Knowledge Infrastructure (CNKI) database, Wanfang database, the Chinese Biomedical Literature database (CBM), and the Chinese Scientific Journals Full Text Database (CQVIP). Another, all the references lists of the included studies will be checked to identify any additional studies. The search terms will be adapted for use with other bibliographic databases in combination with database-specific filters, where these are available. The language will be restricted as English and Chinese. The search strategy of PubMed as an example is shown as follows, and will be modified for other databases use if possible:

1. Psoriasis[Mesh] OR Psoriasis[Title/Abstract] OR Psoriases[Title/Abstract] OR “Pustulosis of Palms and Soles”[Title/Abstract] OR “Pustulosis Palmaris et Plantaris”[Title/Abstract] OR “Palmoplantaris Pustulosis”[Title/Abstract] OR Pustular Psoriasis of Palms and Soles“[Title/Abstract]
2. Hyperuricemia[Mesh] OR Hyperuricemia[Title/Abstract] OR ”Uric Acid“”[Title/Abstract] OR “2,6,8-Trihydroxypurine”[Title/Abstract] OR Trioxopurine[Title/Abstract] OR “Potassium Urate”[Title/Abstract] OR Urate[Title/Abstract] OR “Ammonium Acid Urate”[Title/Abstract] OR “Acid Urate, Ammonium”[Title/Abstract] OR “Urate, Ammonium Acid”[Title/Abstract] OR “Sodium Urate Monohydrate[Title/Abstract] OR ”Monohydrate, Sodium Urate“[Title/Abstract] OR ”Urate Monohydrate, Sodium“[Title/Abstract] OR ”Monosodium Urate Monohydrate“[Title/Abstract] OR ”Monohydrate, Monosodium Urate“[Title/Abstract] OR ”Urate Monohydrate, Monosodium“[Title/Abstract] OR ”Sodium Acid Urate Monohydrate“[Title/Abstract] OR ”Sodium Urate“[Title/Abstract] OR ”Urate, Sodium“[Title/Abstract] OR ”Monosodium Urate“[Title/Abstract] OR ”Urate, Monosodium“[Title/Abstract] OR ”Sodium Acid Urate“[Title/Abstract] OR ”Acid Urate, Sodium“[Title/Abstract] OR ”Urate, Sodium Acid"[Title/Abstract]
3. 1 AND 2

### 2.4 Study selection

**Figure 1:**
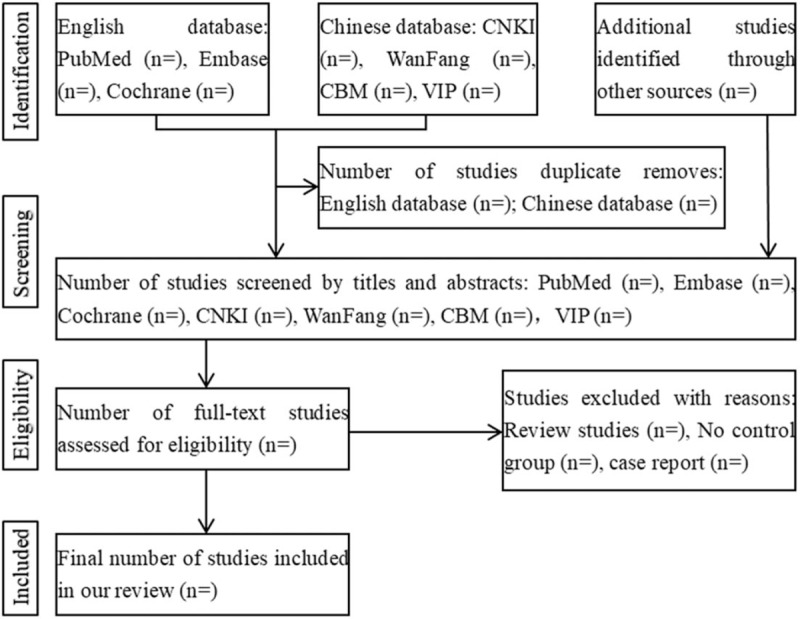
Literature search and study selection flowchart.

Based on the a priori established inclusion and exclusion criteria, titles and abstracts of all the retrieved bibliographic records will be screened through the EndNote X9 literature management software (Thomson Reuters [Scientific] LLC, Philadelphia, PA). Then, full text of all potentially relevant literatures will be retrieved. All the works mentioned earlier will be done independently by 2 authors. Discussions will be used to resolve disagreements between 2 authors or through adjudication of another author. A flow diagram will be used to describe the process according to PRISMA (Fig. 1).

### 2.5 Data extraction and management

The following data will extract from each include study: publication year, 1st author, country where the study was performed, study duration, study design, sample size, participants’ sex, age, ethnicity,country, SUAC levels, risk of hyperuricemia, etc. Two authors will extract data independently using the predefined data extraction form, and disagreements will be resolved by discussion or consult the third author. If the information could not be obtained from the published reports or there are issues to be clarified, we shall contact the lead authors. If the data are still not available, we will perform data synthesis through existing information and address the potential impact of missing data on the pooled results in the discussion parts.

### 2.6 Quality and risk of bias assessment

Two authors will assess the methodologic quality and bias of the included studies using the Newcastle–Ottawa scale (NOS)^[19]^ for observational studies. In NOS, each study will be judged to 8 items and categorized into 3 groups: the comparability of the groups, the selection of the study groups, and the ascertainment of either the exposure or outcome of interest for case–control or cohort studies, respectively. Stars will be awarded for each item, and the highest quality (low risk of bias) studies will be awarded up to 9 stars. We also plan to consider included studies with 0 to 3, 4 to 6, and 7 to 9 stars to represent low, moderate, and high quality, respectively. Any disagreement will be resolved through discussion or consult the 3rd author.

### 2.7 Data synthesis

#### 2.7.1 Description analysis

We plan to use descriptive statistics for study and population characteristics, such as publications year, country, age, sex, etc., to help us facilitate the understanding of the inclusion of the study as a whole.

#### 2.7.2 Standard pairwise meta-analysis

We will perform 2 types of analysis:

1. SUAC levels meta-analysis: the difference in mean SUAC levels between psoriasis patients and healthy controls.
2. Risk of hyperuricemia meta-analysis: the difference of the risk of hyperuricemia between healthy controls and psoriasis patients.

The software of Stata 12.0 will be used to pairwise meta-analysis. The degree of heterogeneity between studies will assess using *I*^2^ tests. *P*-value <.10 or *I*^2^ value >50% will be considered as substantial heterogeneity. In this case, random-effect models will be considered to compute the global mean difference (MD) and risk ratio. For *P*-values >.10 or *I*^2^ <50%, the between-study heterogeneity will not be considered substantial and the fixed-effect models will be used. We will performer network meta-analysis if the prespecified sources of heterogeneity are source population and age. In addition, to investigate others for heterogeneity (such as study design, study quality, severity of psoriasis, etc), we will perform subgroup analysis and meta-regression using prespecified variables and random-effects meta-analysis.

#### 2.7.3 Network meta-analysis

**Figure 2:**
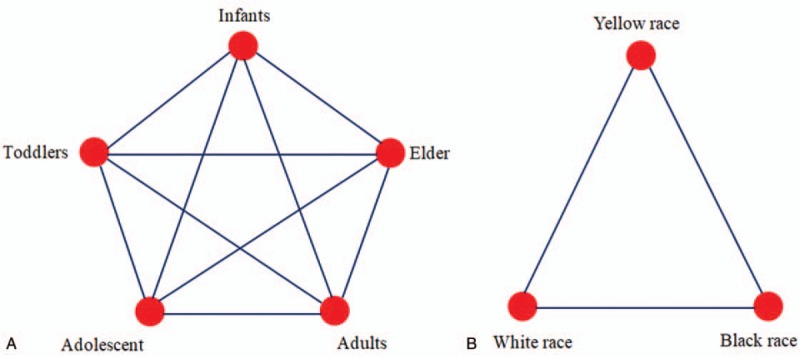
Network of all possible pairwise comparisons between the different age groups (A) and between the different ethnicity groups (B).

Network meta-analysis allows pooling of results derived from direct and indirect evidence.^[20]^ All analyses hereby described will use WinBUGS 1.4.3 software. We will divide the population into infants, toddlers, adolescents, adults, and elder according to their different age. Firstly, we will perform a random-effects network meta-analysis for different age groups and to study which group of patients with psoriasis has the highest SUAC. Figure 2A shows the network of all possible pairwise comparisons between the different age groups. Secondly, we will perform a random-effects network meta-analysis for different ethnicity groups and to study which group of patients with psoriasis has the highest SUAC. Figure 2B shows the network of all possible pairwise comparisons between the different ethnicity groups. Thirdly, if possible we want to perform more random-effects network meta-analysis for different age groups, such as patients from different regions. We used data on intention-to-treat analyses when available. In the network meta-analysis, we accounted for the correlation of effect sizes. Summary MD was presented in a league table. For each outcome, we used the distribution of the ranking probabilities and the surface under the cumulative ranking curves to estimate the relative ranking of the different population. Furthermore, by assuming a common network-specific heterogeneity parameter and estimating predictive intervals, we were able to assess the impact of this heterogeneity on the relative effects with respect to additional uncertainty anticipated in future studies.

Transitivity is the fundamental assumption of indirect comparisons and network meta-analysis. Severity of psoriasis will be considered as potential effect modifiers. Statistical inconsistency can be evaluated with help of local and global approaches. We will use global approaches that are design-by-treatment interaction model and *I*^2^ statistic methods to identify heterogeneity and/or inconsistencies jointly from all possible sources in the network. We will use the comparison-adjusted funnel plot to assess the presence of small-study effects in the network and contour-enhanced funnel plots to investigate whether funnel plot asymmetry is likely to be explained by publication bias. We will also run network meta-regression models that account for differences in the relative effects between smaller and larger studies. We will assess the sensitivity of results for the primary outcome by analyzing only studies considered being at low risk of bias.

### 2.8 Quality of evidence

We are going to adopt the Grading of Recommendations Assessment, Development, and Evaluation (GRADE) approach to assess the quality of evidence of the pooled studies.^[21]^ Limitations of the study, inconsistencies, indirect evidence, inaccuracies, and publication bias will be considered. Levels of evidence quality will be classified into 4 levels: high, moderate, low, or very low.^[22]^

## 3 Discussion

Current research has determined that psoriasis is associated with SUAC levels.^[14]^ However, the specific SUAC level and the incidence of hyperuricemia in patients with psoriasis still need to clarify. This provided the 1st motivation for our study. In addition, the evaluation of more than 2 arm-pairs by traditional meta-analysis is currently limited and difficult. It is unclear that SUAC levels in different groups of psoriasis patients, and there are not enough clinical studies to address this issue. Our study will compare SUCA levels in different groups’ psoriasis patients through network meta-analysis, and we believe our job is very meaningful.

## Author contributions

**Conceptualization:** Hua Du, WenHong Liu.

**Funding acquisition:** Hua Du.

**Methodology:** Yuan Yuan, Ming Liu.

**Project administration:** WenHong Liu, Yuan Yuan.

**Writing – original draft:** Yuan Yuan, Ming Liu.

**Writing – review & editing:** Hua Du, WenHong Liu.
